# Supplementary material for: Sugar Transporters in Plasmodiophora brassicae: Genome-Wide Identification and Functional Verification
Source: Int J Mol Sci. 2022 May 9;23(9):5264. doi: 10.3390/ijms23095264 (PMC9099952; doi:10.3390/ijms23095264)
Supplement: Supplementary file 1 [file ijms-23-05264-s001.zip › Certificate of English Language Editing .pdf]

Certificate of English Language Editing

Manuscript Title:

Sugar Transporters in Plasmodiophora brassicae: Genome-Wide Identification and Functional Verification

Date of Revision:

April 13, 2022

Abstract:

Plasmodiophora brassicae, an obligate intracellular pathogen, can hijack the host's carbohydrates for survival. When the host plant is infected by P. brassicae, a large amount of soluble sugar accumulates in the roots, especially glucose, which probably facilitates the development of this pathogen. Although a complete glycolytic and TCA cycle existed in P. brassicae, very little in-formation about the hexose transport system has been reported. In this study, we screened 17 pu-tative sugar transporters based on information about their typical domains. The structure of these transporters showed a lot of variation compared with that of other organisms, especially the number of transmembrane helices (TMHs). Phylogenetic analysis indicated that these sugar transporters were far from the evolutionary relationship of other organisms and were unique in P. brassicae. The hexose transport activity assay indicated that eight transporters transported glucose or fructose and could restore the growth of yeast strain EBY.VW4000, which was deficient in hexose transport. The expression level of these glucose...

This document certifies that the manuscript listed above was copy edited for English language by LetPub, with regard to grammar, punctuation, spelling, and clarity. All of our language editors are native English speakers with long-term experience in editing scientific and technical manuscripts. We are committed to leveling the playing field for researchers whose native language is not English.

- Documents receiving this certification should be regarded as having undergone professional editorial revision for English language before submission. However, the authors may accept or reject LetPub's suggestions and changes at their own discretion and LetPub does not have editorial control over the submitted documents.
- The language quality of the submitted document is the sole responsibility of the submitting authors subject to those authors' adherence to LetPub's revisions and instruction. LetPub's provision of service does not constitute a guarantee or endorsement of the authors' work herein.
- Neither the research content nor the authors' intended meaning were altered in any way during the editing process.
- If you have any questions or concerns about this edited document, please contact us at support@letpub.com

LetPub
Your Manuscript Editor

LetPub is an author service brand owned and operated by Accdon LLC. Headquartered in the Boston area, we are a full-spectrum author services company with a large team of US-based certified language and scientific editors, ISO 17001 accredited translators, and professional scientific illustrators and animators. We advocate ethical publication practices and are an official member of the Committee on Publication Ethics (COPE).

For more information about our company, services, and partnership programs, please visit www.letpub.com.

© 2022 Accdon, LLC. All Rights Reserved. Tel: 1-781-202-9968 Email: info@accdon.com Address: 400 Fifth Ave, Suite 530, Waltham, MA 02451, United States
