# Supplementary material for: Sugar Transporters in Plasmodiophora brassicae: Genome-Wide Identification and Functional Verification
Source: Int J Mol Sci. 2022 May 9;23(9):5264. doi: 10.3390/ijms23095264 (PMC9099952; doi:10.3390/ijms23095264)
Supplement: Supplementary file 1 [file ijms-23-05264-s001.zip › Table S1.pdf]

Table S1. The primer of *Plasmodiophora brassicae* sugar transporters genes for constructing yeast functional complementation vectors

Gene name	Forward primer	Reverse primer
PbHxt1	ATACCCCAGCCTCGAATGGCTACCTATCAGGGGATG	GTCCAAAGCTGGATCTCAGGCTGAGGTCGTCGAC
PbHxt2	ATACCCCAGCCTCGAATGGCTGTGCTCAGTGCG	GTCCAAAGCTGGATCCTAGCGGGATTTATTCTCTGCATG
PbHxt3	ATACCCCAGCCTCGAATGTCTGCCGAGGAGTCGC	GTCCAAAGCTGGATCTTATTTCCACAATTCCTCGATCTGC
PbHxt4	ATACCCCAGCCTCGAATGACGGCCTCGTACGGG	GTCCAAAGCTGGATCTCAGGCGACAACGTGCTCG
PQ-1	ATACCCCAGCCTCGAATGACGTGCGTCTGTATTCC	GTCCAAAGCTGGATCTCAAACAGCCGAGGAATGC
PQ-2	ATACCCCAGCCTCGAATGATGGCGAATGGTCGA	GTCCAAAGCTGGATCCTATTCGGGGCGCCTTTCA
PQ-3	ATACCCCAGCCTCGAATGTCGCCTGTGTCCTGC	GTCCAAAGCTGGATCCTAGTATTGCGACTCCAGGTC
PQ-4	ATACCCCAGCCTCGAATGATGCAATGTGTATGCAAGG	GTCCAAAGCTGGATCTCATTGCTTCTCGATGTCCCTCG
PQ-5	ATACCCCAGCCTCGAATGGCGTGGTGTGAATGCG	GTCCAAAGCTGGATCTTACTTTGATTGCCGGTACATCC
PQ-6	ATACCCCAGCCTCGAATGTCGTGCGACCTGCCG	GTCCAAAGCTGGATCTTATCCCGACGGCATGGG
PQ-7	ATACCCCAGCCTCGAATGGGGACCGCTCCGCGC	GTCCAAAGCTGGATCTCAGATGGCGACCGGGAGG
PQ-8	ATACCCCAGCCTCGAATGGAGAGAGCACATACGTATGGG	GTCCAAAGCTGGATCTTATACATCGGGTGTATCAGGTGCG
PQ-9	ATACCCCAGCCTCGAATGTCGGCCTCGCATGTG	GTCCAAAGCTGGATCTCATGGATGCTTCGGCGC
PQ-10	ATACCCCAGCCTCGAAAAGAGGAAAAAATTGTTAGGGGT	GTCCAAAGCTGGATCACTTTCACAAGTATATGCATCATCT
PQ-11	ATACCCCAGCCTCGAATGCCTCCGTATAACCGG	GTCCAAAGCTGGATCTCATAGTGTAGGCGAAGGCG
PQ-12	ATACCCCAGCCTCGAATGGACGCCGTCGTTGTCG	GTCCAAAGCTGGATCAATGAAATCCTTGTGCGCTAGGC
PQ-13	ATACCCCAGCCTCGAATGGACGTCGTCCGCACG	GTCCAAAGCTGGATCCTAGTCCTTCTTCGTGGTCTTTGAC
PQ-14	ATACCCCAGCCTCGAATGGGGATCGCTCCGCGC	GTCCAAAGCTGGATCTCAGATGGCGACCGGGAGG
