# Supplementary material for: Sugar Transporters in Plasmodiophora brassicae: Genome-Wide Identification and Functional Verification
Source: Int J Mol Sci. 2022 May 9;23(9):5264. doi: 10.3390/ijms23095264 (PMC9099952; doi:10.3390/ijms23095264)
Supplement: Supplementary file 1 [file ijms-23-05264-s001.zip › Table S2.pdf]

Table S2. The quantitative real-time PCR primer for sugar transporter genes of *Plasmodiophora brassicae*

Gene name	Forward primer	Reverse primer
PbHxt3	GCCGTTGCCGTTTACTATTC	CGCACTGAACTGGGATAGAT
PQ-3	GTTCTGCGGTATCCAGTTCAT	GTGCTTGGCGAATAGGTAGAG
PQ-4	CTGTGACTACGAGAGCGATAAC	GGAATACGCGAGAAGAAGTACA
PQ-5	GCTCGAATATGTCCCGTCAAT	CAATGCTTGTTGCGGTAGTTG
PQ-7	CCAGCAAGTCGTCGTCAATTA	GAAGATGGCGTAGAACAGGAAC
PQ-9	CCTACGCCCAAACGATGATA	GTACGACGACACGGAATAGTG
PQ-12	TCTTCGCTGTTGGCATGT	ATACGGTCGTTGCCATGTAG
PQ-13	ACTTTGTCCTCGCATTCGT	AGTCCTTCTTCGTGGTCTTTG
Pbactin	GGGACATCACCGACTACCTG	ACTGCTCCGAGTTGGACATC
