# Supplementary figures and images for: Sugar Transporters in Plasmodiophora brassicae: Genome-Wide Identification and Functional Verification
Source: Int J Mol Sci. 2022 May 9;23(9):5264. doi: 10.3390/ijms23095264 (PMC9099952; doi:10.3390/ijms23095264)

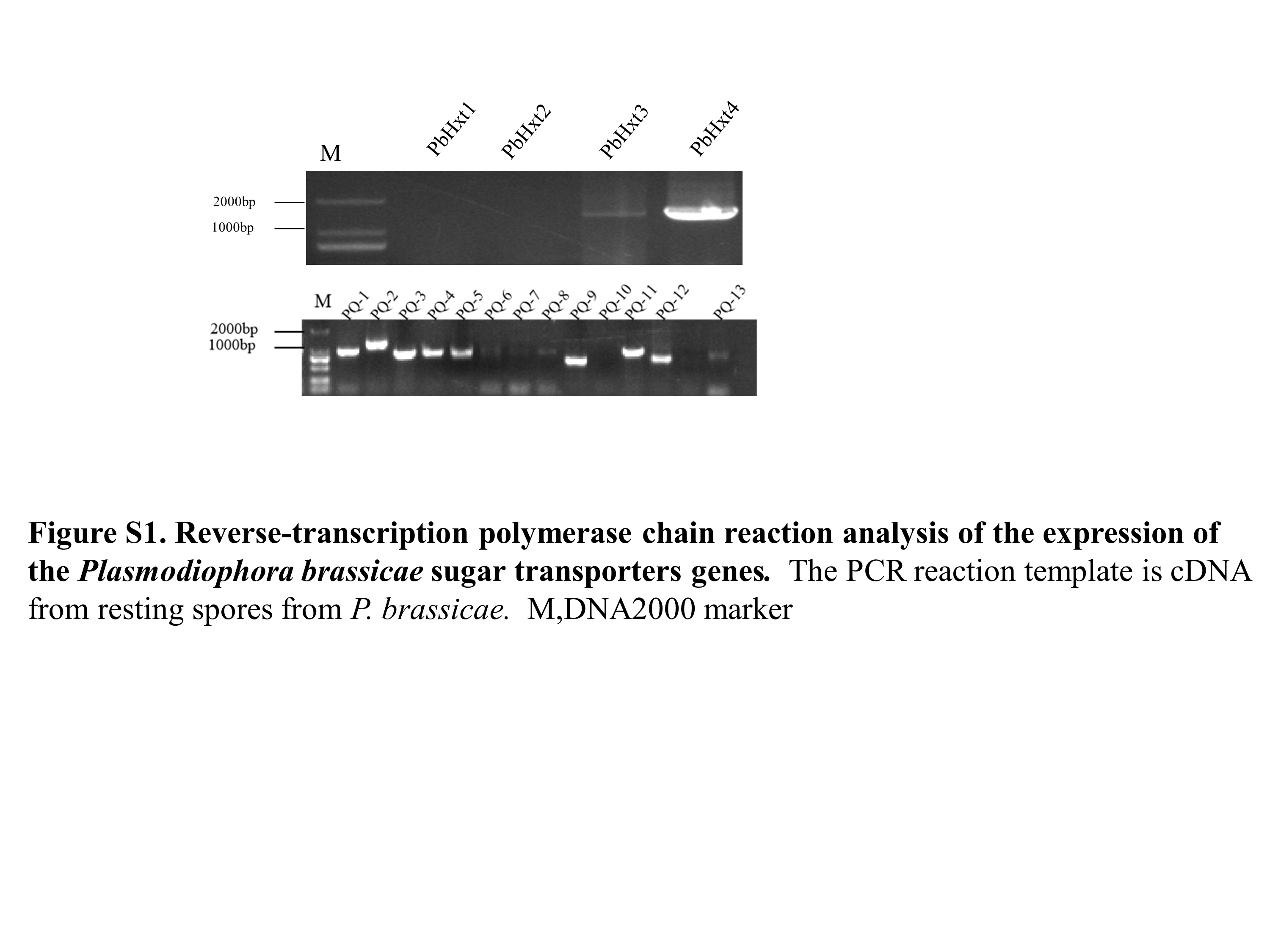

Supplement: Supplementary file 1 [file ijms-23-05264-s001.zip › FigureS1.TIF]

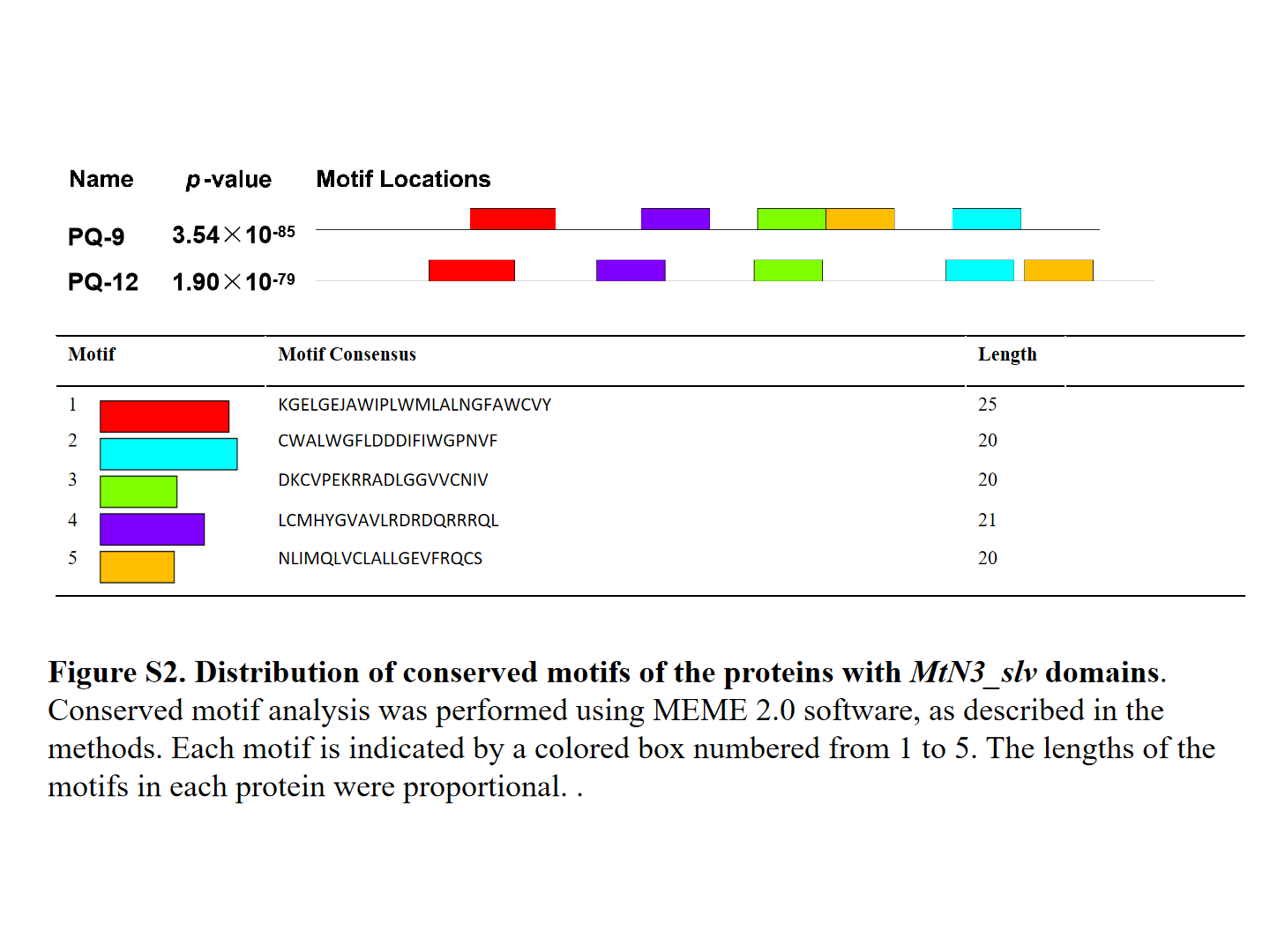

Supplement: Supplementary file 1 [file ijms-23-05264-s001.zip › FigureS2.tif]
